# Diabetes Mellitus is a Risk Factor for Developing CMV Retinitis in Solid Organ Transplant Recipients: A Case-control Study

**DOI:** 10.1093/ofid/ofag056

**Published:** 2026-02-06

**Authors:** Dilpreet K Bharaj, Shanil Dhanji, Elena Gurung, Gale Ladua, Christopher F Lowe, Nancy Matic, Justin Gill, Allison Mah, Alissa J Wright, David Ta Kim, Kaivon Pakzad-Vaezi, Sara Belga

**Affiliations:** Faculty of Medicine, University of British Columbia, Vancouver, British Columbia, Canada; Department of Ophthalmology and Visual Sciences, University of British Columbia, Vancouver, British Columbia, Canada; Department of Ophthalmology and Visual Sciences, University of British Columbia, Vancouver, British Columbia, Canada; Department of Medicine, Division of Infectious Diseases, University of British Columbia, Vancouver, British Columbia, Canada; Division of Medical Microbiology and Virology, St.Paul's Hospital, Vancouver, British Columbia, Canada; Department of Pathology and Laboratory Medicine, University of British Columbia, Vancouver, British Columbia, Canada; Division of Medical Microbiology and Virology, St.Paul's Hospital, Vancouver, British Columbia, Canada; Department of Pathology and Laboratory Medicine, University of British Columbia, Vancouver, British Columbia, Canada; Department of Medicine, Division of Nephrology, University of British Columbia, Vancouver, British Columbia, Canada; Department of Medicine, Division of Infectious Diseases, University of British Columbia, Vancouver, British Columbia, Canada; Department of Medicine, Division of Infectious Diseases, University of British Columbia, Vancouver, British Columbia, Canada; Department of Ophthalmology and Visual Sciences, University of British Columbia, Vancouver, British Columbia, Canada; Department of Ophthalmology and Visual Sciences, University of British Columbia, Vancouver, British Columbia, Canada; Department of Medicine, Division of Infectious Diseases, University of British Columbia, Vancouver, British Columbia, Canada; Immunity and Infection Research Centre, Vancouver Coastal Health Research Institute, Vancouver, British Columbia, Canada

**Keywords:** cytomegalovirus (CMV), retinitis, risk factors, solid organ transplantation (SOT)

## Abstract

**Background:**

The incidence of cytomegalovirus retinitis (CMVR) in solid organ transplant (SOT) recipients may be increasing due to improved long-term survival post-transplant. However, the epidemiology of CMVR after SOT is not well described. We therefore aimed to determine the incidence of CMVR and identify risk factors for its development post-SOT.

**Method:**

Case-control study at a 1:4 ratio between January 1, 2012 and March 31, 2024. Cases were SOT recipients at 1 of 2 British Columbia transplant centres diagnosed with CMVR clinically or through ocular fluid viral PCR. Controls were matched on transplant organ, year, and center. Univariable and multivariable analyses were performed using conditional logistic regression models.

**Results:**

Out of 5877 SOT recipients followed during the study period, there were 16 CMVR cases with an incidence rate of 5.3 per 10 000 person-years. Fourteen eligible cases of CMVR were matched to 56 controls. Cases were older than controls (median age 60.9 vs 42.6 years) and more likely to have diabetes mellitus (DM) (9 [64.3%] vs 9 [16.4%]), chronic heart disease (4 [28.6%] vs 4 [7.3%]), and CMV DNAemia any time post-SOT (12 [85.7%] vs 19 [35.2%]). Most received kidney transplants (50.0%), and the median time to CMVR was 3.4 years. After adjusting for age at transplant, DM, chronic heart disease, CMV DNAemia, and significant lymphopenia, only DM was associated with an increased odds of CMVR (odds ratio 16.5 [95% confidence interval, 1.12–243.8]).

**Conclusions:**

CMVR is a rare and late complication post-SOT. DM was independently associated with increased odds of CMVR post-SOT.

## Teaser

In this case-control study in SOT recipients, the incidence of CMV retinitis was found to be 5.3 per 10 000 person-years, with a cumulative incidence of 0.3%. DM was independently associated with increased odds of CMV retinitis post-transplant.

Cytomegalovirus (CMV) infection is a common complication after solid organ transplantation (SOT) and can progress to end-organ diseases such as pneumonia, gastritis, hepatitis, and retinitis [1]. Approximately 50% of SOT recipients (SOTr) develop symptomatic CMV infection, with donor-positive and recipient-negative (D+/R−) CMV serostatus being a major risk factor for developing CMV disease [2, 3]. CMV retinitis (CMVR) is a severe, vision-threatening disease usually seen in immunocompromised patients. CMVR is also the leading cause of blindness in patients with human immunodeficiency virus (HIV) and, therefore, has mostly been studied in this population, with resultant ocular screening guidelines being developed in the presence of low CD4 counts [4]. Although rare in the SOT setting, there is an increased incidence of CMVR in SOTr because of prolonged survival after organ transplantation due to advances in post-transplant care [4].

A case series of hematopoietic stem cell transplant (HSCT) and SOTr who developed CMVR by Eid et al, presented descriptive findings regarding the clinical characteristics and ophthalmological findings in these patients. In this study, 9 cases were identified in a 15-year period. The median age of cases at the time of CMVR was 58 years, and most patients had received kidney transplants (*n* = 5). Hypertension was identified as a comorbidity in 5 patients, whereas diabetes mellitus (DM) was identified in 4 patients. Finally, only 4 patients had CMV DNAemia, and 4 patients had developed additional CMV end-organ disease (hepatitis *n* = 2 and pneumonitis *n* = 2). This study highlighted the irreversibility of vision loss associated with CMVR as a post-transplant complication, and a delay in diagnosis as compared with other CMV end-organ diseases [5].

Another retrospective cohort study of 39 transplant recipients (18 SOT and 21 HSCT) with CMV DNAemia referred to ophthalmology by Son et al compared the clinical features of CMVR between SOT and HSCT. CMVR developed in 8.7% of patients with CMV DNAemia following SOT in 10 years, with a median time to CMVR of 5.8 months. Key differences described between SOT and HSCT included mortality being significantly higher in the HSCT group, visual acuity being lower in the SOT group, and most patients requiring vitrectomy for retinal detachment having received SOT [6].

Most recently, a retrospective cohort study by Scherger et al described the occurrence of CMVR in high-risk SOTr with CMV DNAemia who underwent routine ophthalmologic examination. Out of the 38 patients evaluated, 13.2% developed CMVR during the 4-year study period. The mean time to CMVR from transplantation was 281 days. In this study, most patients lacked ocular symptoms at the time of diagnosis [7].

Given that prior studies were mostly descriptive, the true incidence of CMVR post-SOT remains largely unknown. Additionally, since recognition of clinical risk factors to allow for early diagnosis and treatment of CMVR is key in preventing permanent retinal damage and vision loss, we aimed to calculate the incidence rate of CMVR and perform a retrospective case-control study to identify risk factors for developing CMVR in the SOT population.

## METHODS

### Study Design

This is a retrospective case-control study of SOTr in the province of British Columbia (BC), Canada, from January 1, 2012 to March 31, 2024. The 2 adult transplant centers in BC are Vancouver General Hospital (VGH) and St. Paul's Hospital (SPH), which serve a population of ∼5.7 million people. We classified CMVR cases as patients who had received SOT at either 1 of the 2 centers and were diagnosed with CMVR based on clinical criteria, or when available, confirmed by a positive ocular fluid PCR, in accordance with consensus definitions and guidelines [8, 9]. Cases were matched to controls at a 1:4 ratio based on transplant organ, year, and center, and had follow-up data available from the time of transplant to the time of their matched case's CMVR diagnosis.

This study was approved by the University of British Columbia Clinical Research Ethics Board (H23-01580).

### Patient Consent Statement

The requirement for informed consent was waived due to the retrospective design of the study.

### Patient Population

To capture all the CMVR cases within the province, CMVR cases were identified through a database obtained from a centralized provincial reference laboratory at SPH, where all CMV ocular fluid PCR requests for adult patients in BC are performed. To capture CMVR cases that were diagnosed clinically and may not have had CMV ocular fluid PCR testing done at all, we completed a data search of the electronic medical record system used at the 2 transplant centers (VGH and SPH) using Canadian Classification of Health Interventions (CCI) and International Classification of Diseases (ICD-10) diagnostic codes through the Data and Analytics team at Vancouver Coastal Health. SOTr were referred to ophthalmology at the discretion of the treating clinicians, mostly transplant or transplant infectious diseases physicians. According to institutional practices, ocular fluid is sent for CMV PCR testing when CMVR is suspected, including SOTr presenting with new visual disturbances or changes in visual acuity. In rare situations, when retinal findings were highly characteristic, and SOTr concurrently had CMV DNAemia, ocular fluid was not obtained, and a CMVR diagnosis was made clinically. Of note, ophthalmological evaluation is not routinely performed for SOTr with CMV DNAemia or extra-ocular CMV disease, unless there are ocular concerns.

Exclusion criteria for both cases and controls included not having received SOT, receiving SOT at a foreign or out-of-province transplant center (these individuals were included in the incidence calculation but not in the case-control analysis), and CMVR cases without a formal diagnosis by an experienced ophthalmologist based on the Standardization of Uveitis nomenclature classification criteria [10]. Potential CMVR cases identified through diagnostic codes who had a negative CMV ocular fluid PCR result were excluded, given the high sensitivity (≥90.0%) of CMV ocular fluid PCR for diagnosis of CMVR [11].

### Data Collection

Data collection was completed through retrospective chart review of the electronic medical record systems. We collected demographic data, information regarding SOT, post-transplant complications, comorbidities, and laboratory results for cases and controls, and additionally collected ophthalmological characteristics for cases.

Post-transplant complications were recorded anytime between the date of transplant and the date of CMVR diagnosis for cases and their matched controls. The type of maintenance immunosuppression, concurrent CMV therapy, and laboratory values were recorded at the time of CMVR diagnosis (+/−3 months) for cases and their matched controls. All ophthalmological data were collected at the time of presentation of CMVR.

CMV PCR on ocular fluid was conducted on a laboratory-developed qualitative PCR test (LDT) targeting the CMV glycoprotein B (UL55) gene sequence. Samples were extracted on either the MagNA Pure Compact or MagNA Pure 24 system (Roche Diagnostics, Pleasanton, CA), and DNA amplified by real-time PCR on the Light Cycler 480 II (Roche). CMV DNAemia (viral load) at the time of CMVR diagnosis was recorded as IU/mL for all patients. Before June 13, 2017, CMV DNAemia was performed on a validated LDT, as previously described, and then transitioned to the cobas® CMV assay on the cobas® 6800 platform (Roche) [12]. CMV DNAemia tested on the LDT was reported as undetectable, <1000 copies/mL (1–999) or the numerical value (≥1000 copies/mL). For this study, values reported on the LDT in copies/mL were converted to IU/mL using the conversion factor, 1.211, based on our laboratory validation. On the cobas® CMV, results of <35 IU/mL were coded as 0 and ≥35 IU/mL as the respective numerical value. Ocular fluid PCR was qualitative only, given the limitations of the very low fluid volume typically available from the anterior chamber of the eye.

### Statistical Analysis

Categorical variables were expressed as proportions and compared by the χ^2^ test. Continuous variables were expressed as means if normally distributed and medians if not normally distributed and compared by *t*-test or Wilcoxon rank-sum testing.

For the incidence calculation, time-at-risk was defined from time in years from transplant or first follow-up in BC to the date of last follow-up (or end of study, whichever came first) for controls, and time from transplant or first follow-up in BC to the date of CMVR diagnosis for cases. Patients who received SOT outside of BC were not included in the case-control analysis and were, therefore, not included in any risk factor analyses. However, to ensure an accurate population-level estimate of CMVR incidence, these patients were included in our incidence calculation, as there are many SOTr who received SOT outside of BC but later relocated to BC and have complete longitudinal follow-up within the provincial transplant registry. The start of time-at-risk for patients who received SOT outside of BC was their first follow-up visit in BC to avoid an immortal time bias, ensuring appropriate alignment of at-risk time with the period during which CMVR could be detected within provincial systems.

Conditional logistic regression models were created to conduct univariable and multivariable analyses assessing the significance of varying clinical characteristics between cases and controls. Significance level was set at a two-sided *P* value <.05. Select variables with *P* < .05 on univariable analysis were included in the final multivariable analysis. All analyses were performed with Stata/SE (StataCorp LLC, version 18.0, College Station, TX).

## RESULTS

### Incidence

Between January 1, 2012 and March 31, 2024, there were 5877 SOTr followed in BC for a total of 29 939.9 person-years, specifically 3793 kidney (+/− combined with pancreas), 1129 liver, 605 lungs (including 1 combined heart–lung), 328 hearts, and 22 pancreas alone transplant recipients. There were 16 SOTr with CMVR, resulting in an incidence rate of 5.3 per 10 000 person-years and a cumulative incidence of 0.3%.

### Patient Demographics

**Figure 1.**
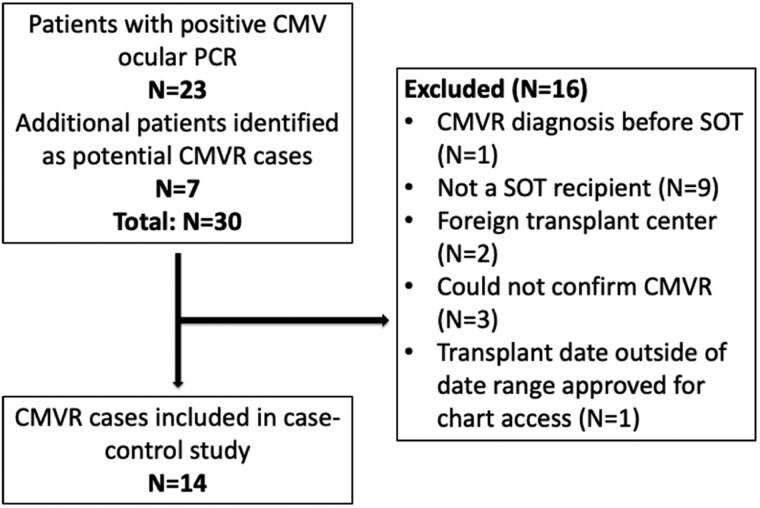
CONSORT diagram of CMVR case identification

A total of 30 potential CMVR cases were identified. However, 16 patients were excluded for various exclusion criteria, including 2 SOTr transplanted at a foreign center (Figure 1).

We identified 14 eligible cases (57.1% male) of CMVR, with 56 matched controls (53.6% male) (Table 1). Most cases and controls received kidney transplants (50.0%), with the remainder receiving heart (21.4%), lung (21.4%), and liver transplants (7.1%).

**Table 1.** Baseline Demographic and Clinical Characteristics of CMV Retinitis Cases and Matched Controls

Factor		Cases	Controls	*P* Value
*N* (%)		14	56	-
Age at transplant, mean (SD)		60.9 (13.3)	42.6 (15.6)	<.001
Sex	Male	8 (57.1%)	30 (53.6%)	.81
	Female	6 (42.9%)	26 (46.4%)	
Transplant organ	Kidney	7 (50.0%)	28 (50.0%)	1.00
	Liver	1 (7.1%)	4 (7.1%)	
	Lung	3 (21.4%)	12 (21.4%)	
	Heart	3 (21.4%)	12 (21.4%)	
CMV D/R status	CMV D−/R−	1 (7.1%)	6 (10.7%)	.69
	CMV D+/R−	1 (7.1%)	16 (28.6%)	.094
		12 (85.7%)	34 (60.7%)	.078
Previous transplant		2 (14.3%)	7 (12.5%)	.86
Post-transplant complications	Acute rejection	2 (14.3%)	19 (33.9%)	.15
	Graft dysfunction	2 (14.3%)	4 (7.1%)	.39
	CMV DNAemia	12 (85.7%)	19 (35.2%)	<.001
Comorbidities	Diabetes mellitus	9 (64.3%)	9 (16.4%)	<.001
	Chronic heart disease	4 (28.6%)	4 (7.3%)	.026
Absolute lymphocyte count (x 10^9/L), median (IQR)		1.0 (0.6, 1.3)	1.5 (1.1, 2.0)	.003
Significant lymphopenia (ALC ≤ 0.6 × 10^9/L)		3 (21.4%)	1 (1.8%)	.005
Induction immunosuppression	ATG	6 (42.9%)	9 (17.3%)	.043
	Basiliximab	7 (50.0%)	39 (75.0%)	.071
	Methylprednisolone	13 (92.9%)	50 (96.2%)	.60
Maintenance immunosuppression	Mycophenolate/	11 (78.6%)	40 (71.4%)	.59
	mycophenolic acid	…		
	Tacrolimus	12 (85.7%)	53 (94.6%)	.25
	Azathioprine	0 (0.0%)	8 (14.3%)	.13
	Sirolimus	3 (21.4%)	4 (7.1%)	.11
	Prednisone	8 (57.1%)	34 (60.7%)	.81
	None	1 (7.1%)	0 (0.0%)	.044

Data are presented as median (interquartile range [IQR]) or mean (standard deviation [SD]) for continuous measures, and *n* (%) for categorical measures.

Abbreviations: ALC, absolute lymphocyte count; ATG, antithymocyte globulin; CMV, cytomegalovirus; D/R, donor/recipient; Unk, unknown.

The median age of cases at transplant was significantly higher (60.9 years) than that of controls (42.6 years) (*P* < .001). Cases were more likely to have DM at transplant (64.3% vs 16.4%, *P* < .001), chronic heart disease at transplant (28.6% vs 7.3%, *P* = .026), to have lower median absolute lymphocyte count (ALC) (1.0 × 10^9/L vs 1.5 × 10^9/L, *P* = .003) and significant lymphopenia defined as ALC ≤0.6 × 10^9/L (21.4% vs 1.8%, *P* = .005) at the time of CMVR diagnosis, and the occurrence of CMV DNAemia anytime between transplant and before CMVR diagnosis (85.7% vs 35.2%, *P* < .001). Cases were also more likely to have received antithymocyte globulin (ATG) as induction immunosuppression compared with controls (42.9% vs 17.3%, *P* = .043) (Table 1).

Eight out of 14 cases had a detectable but low CMV viral load at the time of CMVR diagnosis. The median CMV viral load for cases at this time-point was 48 IU/mL (interquartile range [IQR] 0–574 IU/mL), which was higher than that of the controls, 0 IU/mL (IQR 0–0). However, the mean CMV DNAemia was higher in the controls (96 108 IU/mL, standard deviation [SD] 559 047) versus the cases (4046.4, SD 14 410).

Median time to CMVR was 3.4 years (IQR, 2.0–7.9 years).

### Univariable Analyses

The unadjusted odds ratios (OR) (with 95% confidence intervals [CI]) for age, DM, chronic heart disease, ALC, significant lymphopenia and CMV DNAemia were 1.07 (95% CI, 1.03–1.12), 12.4 (95% CI, 2.56–59.5), 6.0 (95% CI, 1.06–33.9), 0.22 (95% CI, .06–.79), 12.0 (95% CI, 1.25–115.4), and 13.1 (95% CI, 1.68–101.7), respectively (Table 2).

**Table 2.** Univariable and Multivariable Logistic Regression Analyses of Potential Risk Factors for Developing CMVR Post-SOT

Factor		Unadjusted OR (95% CI)	*P* Value	Adjusted OR (95% CI)	*P* Value
Age at transplant		1.07 (1.03–1.12)	.002	1.03 (.97–1.09)	.316
Sex		.85 (.25–2.93)	.803	…	…
CMV D/R status	CMV D−/R−	Ref.	…	…	…
	CMV D+/R−	.22 (.03–1.73)	.150	…	
		3.43 (.74–15.9)	.115	…	…
Post-transplant complications	Acute rejection	.27 (.05–1.51)	.137	…	…
	Graft dysfunction	2.17 (.35–13.5)	.404	…	…
	Chronic kidney disease post-transplant	1.83 (.47–7.07)	.383	…	…
	CMV DNAemia	13.1 (1.68–101.7)	.014	8.21 (.57–118.9)	.123
Comorbidities	Chronic kidney disease pretransplant	1.78 (.26–12.5)	.560	…	…
	Chronic liver disease	2.45 (.14–42.6)	.539	…	…
	Chronic heart disease	6.00 (1.06–33.9)	.043	18.3 (.27–1243.5)	.177
	Diabetes mellitus	12.4 (2.56–59.5)	.002	16.5 (1.12–243.8)	.041
	Solid malignancy	3.42 (.67–17.4)	.138	…	…
	Autoimmune disease	1.44 (.23–9.04)	.696	…	…
Chronic kidney disease (total)		2.03 (.43–9.56)	.370	…	…
Laboratory values at time of CMVR diagnosis	Significant lymphopenia (ALC ≤.6 × 10^9/L)	12.0 (1.25–115.4)	.031	4.74 (.65–348.4	.478
	CMV DNAemia (numerical)	1.00 (1.00–1.00)	.677	…	…
Induction Immunosuppression	ATG	7.13 (.77–66.4)	.084	…	…
	Basiliximab	.24 (.04–1.31)	.099	…	…
Maintenance Immunosuppression	Mycophenolate/mycophenolic acid	1.51 (.35–6.44)	.581	…	…
	Tacrolimus	.23 (.02–2.73)	.246	…	…
	Sirolimus	4.55 (.71–29.3)	.111	…	…
	Prednisone	.69 (.10–4.50)	.695	…	…
	Triple maintenance	.54 (.12–2.45)	.421	…	…
	Antimetabolite	.60 (.13–2.78)	.509	…	…
	Calcineurin inhibitors	.23 (.02–2.73)	.246	…	…

Abbreviations: ALC, absolute lymphocyte count; ATG, antithymocyte globulin; CI, confidence interval; CMV, cytomegalovirus; CMVR, cytomegalovirus retinitis; D/R, donor/recipient; OR, odds ratio; Ref., reference group; SOT, solid organ transplant; Unk, unknown.

### Multivariable Analysis

Out of age, DM, chronic heart disease, significant lymphopenia, and CMV DNAemia, DM was the only variable independently associated with an increased odds of CMVR (OR 16.5 [95% CI, 1.12–243.8]; *P* = .041) on multivariable analysis (Table 2).

### Ophthalmological Characteristics of CMV Retinitis Cases

Eleven cases (78.6%) had unilateral, and 3 (21.4%) cases had bilateral CMVR. Most cases had symptoms at the time of presentation, with blurred vision being present in the majority (Table 3). Visual acuity was decreased in 13 out of 14 cases, ranging from 20/25 to no light perception in the affected eye(s). Ten cases were treated with intravitreal antiviral therapy (71.4%), and 8 patients were treated with oral, systemic antiviral therapy (57.1%). Only 2 out of 14 cases underwent vitrectomy for retinal detachment (Table 3). Thirteen cases had a positive CMV ocular fluid PCR result (92.9%). Most cases had existing ocular comorbidities. Only 2 cases were identified to have recurrence of CMVR after initial treatment. An additional 2 cases were identified to have retinal detachment after their CMVR diagnosis. Ophthalmological findings of CMVR cases are summarized in Table 3.

**Table 3.** Ophthalmological Characteristics of CMV Retinitis Cases

CMVR Cases	Location	Symptoms	Visual Acuity	IOP	Disease Activity	Treatment	Time Until Resolution (m)	CMV Ocular Fluid PCR Results	Ocular Comorbidities	Ocular Complications
1	Left	Unk	NLP	20	Unk	Intravitreal ganciclovir	Unk	Pos	PDR	Unk
2	Left	Blurred vision, pain	CF	52	Panuveitis: KP, trace AC cells, retinitis, vasculitis	Intravitreal ganciclovir, oral valganciclovir	3.5	Pos	CRVO	Subhyaloid hemorrhage, vitreous hemorrhage
3	Left	Blurred vision, floaters, fevers	HM	20	Panuveitis: KP, anterior cells 2+, retinitis, vasculitis	Intravitreal foscarnet, oral valganciclovir	4	Pos	Severe NPDR	CRAO/peripheral ischemia, recurrence both eyes 6 m later
4	Right	Blurred vision	20/25	26	Panuveitis: KP, anterior cells 2+, vitreous cells 2+, retinitis	Intravitreal foscarnet, oral valganciclovir	1	Pos	PDR with bilateral vitrectomy and PRP	Atrophic hole in area of resolved retinitis
5	Left	Unk	Unk	Unk	Unk	Unk	Unk	Pos	CRVO	Unk
6	Right	Blurred vision, floaters	20/30	24	Retinitis	Oral valacyclovir, then oral valganciclovir	6	Pos	Unk	BRVO
7	Bilateral	Blurred vision	OU 20/30	OD 15 OS 16	Right anterior and intermediate uveitis: 1 + cells, haze; left panuveitis: KP, anterior cells 2+, vitreous cells 2+, retinitis	Intravitreal foscarnet, oral valganciclovir (switched to intravitreal ganciclovir later)	1	Pos	Moderate NPDR	CRVO (requiring intravitreal bevacizumab), pancytopenia, recurrence both eyes 4 m later, died 14 m later
8	Right	Unk	CF	Unk	Panuveitis: KP, no AC cells, vitritis, retinitis	Unk	Unk	Pos	Unk	Unk
9	Left	Blurred vision	HM	18	Panuveitis: KP, anterior cells 2+, vitreous cells 2+, retinitis	Intravitreal foscarnet, oral valganciclovir	1	Pos	Unk	CRVO
10	Right	Blurred vision, headache	CF	21	Panuveitis: KP, anterior cells 2+, vitreous cells 2+, retinitis	Intravitreal foscarnet, oral valganciclovir	1	Pos	Moderate NPDR	Unk
11	Right	Blurred vision	HM	27	Retinitis	Unk	Unk	Pos	PDR	Unk
12	Right	Blurred vision, floaters	CF	13	Panuveitis: KP, anterior cells 2+, vitreous cells 2+, retinitis	Intravitreal foscarnet, oral famvir (switched to oral valganciclovir later)	2	Pos	Unk	Died 13 m later
13	Bilateral	Blurred vision	OD 20/200 OS CF	OD 12 OS 8	Panuveitis: KP, anterior cells 2+, vitreous cells 2+, subretinal infiltrate	Intravitreal foscarnet, vitrectomy for retinal detachment	Unk	N/A; ocular fluid not collected	ERM, mild NPDR, CSR`	Bilateral inferior retinal detachments
14	Bilateral	Blurred vision	OD 20/20 OS 20/200	OD 14 OS 15	Vitritis, retinitis OU	Intravitreal foscarnet, vitrectomy for retinal detachment	4	Pos	Unk	Right inferior retinal detachment, CME

Abbreviations: AC, anterior chamber; BRVO, branch retinal vein occlusion; CF, counting fingers; CME, cystoid macular edema; CMVR, cytomegalovirus retinitis; CRAO, central retinal artery occlusion; CRVO, central retinal vein occlusion; CSR, central serous retinopathy; ERM, epiretinal membrane; HM, hand motion; KP, keratic precipitates; Neg, negative; NLP, no light perception; NPDR, nonproliferative diabetic retinopathy; OD, right eye; OS, left eye; OU, both eyes; PDR, proliferative diabetic retinopathy; Pos, positive; PRP, panretinal photocoagulation; Unk, unknown.

Proliferative diabetic retinopathy (PDR) was identified as a comorbidity in 3 out of the 14 cases (21.4%), and nonproliferative diabetic retinopathy (NPDR) was identified in 4 out of 14 cases (28.6%) (Table 3). Of the 9 CMVR cases with known diabetes, 6 patients had diabetic retinopathy, ranging from mild NPDR to PDR. Interestingly, 1 case without documented diabetes on their past medical history had mild NPDR. However, this case was noted to have developed post-transplant diabetes as a complication post-SOT.

## DISCUSSION

In this single-center study, spanning 12 years and 5877 SOTr, the incidence rate of CMVR was 5.3 per 10 000 person-years, and the cumulative incidence was 0.3%. Our final study population included 14 eligible cases of CMVR matched to 56 controls. The incidence of CMVR in our study was lower at 0.3%, compared with 8.7% in the study by Son et al spanning 10 years and 13.2% in the study by Scherger et al spanning 4 years [6, 7]. We believe the incidence of CMVR in these two studies was likely overestimated due to selection bias, as only patients with CMV DNAemia undergoing ophthalmological evaluation were studied. Our study's strength is that it represents the incidence of CMVR in all patients receiving SOT in BC. The median time to CMVR post-transplant was found to be 3.4 years in this study, which occurred later than previously reported [6, 7].

Cases were more likely to have increased age at transplant, DM, chronic heart disease, significant lymphopenia at the time of CMVR diagnosis, and CMV DNAemia anytime between SOT and CMVR diagnosis and were identified as potential risk factors for developing CMVR post-SOT. Most notably, only DM, pre-existing at the time of transplant, was independently associated with increased odds of CMVR in this population, which is being described for the first time. The presence of significant lymphopenia (ALC ≤0.6 × 10^9/L) at the time of CMVR diagnosis, rather than the median ALC, was studied in the multivariable analysis, as ALC below a threshold of 610 cells/μL has been reported in the literature to be associated with the development of CMV infection [13, 14]. Although most cases had CMV DNAemia in our study, this was mostly low-level, which might not have been detected depending on the sensitivity of the assay. Furthermore, previous studies describing CMVR in SOT were limited to small sample sizes with absent controls, with some studies combining SOT and HSCT populations, which likely have inherently different risk factors.

This novel finding of DM being independently associated with increased odds of CMVR post-SOT prompts the consideration of closer surveillance for CMVR post-SOT in patients with DM. We hypothesize that diabetic retinopathy, by means of chronic inflammation and damage, might facilitate access of CMV into the retina. The current Diabetes Canada guidelines encourage screening ocular exams for patients who have not already developed diabetic retinopathy at diagnosis and then annually for patients with type 1 diabetes or every 1–2 years for patients with type 2 diabetes [15]. Although the findings of this study may necessitate a reconsideration of these guidelines for SOTr with DM, specific surveillance for CMVR may be difficult to justify while acknowledging that the diagnosis of CMVR remains rare despite the high prevalence of diabetes in the transplant population. However, it may be prudent to inform the ocular specialist performing diabetic retinopathy screening of the SOT status of their patient.

CMV DNAemia at any time post-transplant before CMVR diagnosis was found to be a statistically significant clinical risk factor for developing CMVR post-SOT on univariable analysis. Although most CMVR cases also had detectable CMV DNAemia at the time of CMVR diagnosis, the median CMV viral load value was <100 IU/mL. Scherger et al described a similar finding in which CMVR cases had low viral loads at the time of CMVR diagnosis but had preceding higher viral loads [7]. These results demonstrate that the presence of CMV DNAemia anytime post-SOT is likely necessary to develop CMVR. Therefore, CMV DNAemia may need to be considered in conjunction with a history of DM to inform which SOT patients may be at risk for developing CMVR.

The low or negative CMV DNAemia at the time of CMVR diagnosis may suggest compartmentalization, as has been previously postulated and described, similarly to gastrointestinal disease [16, 17]. Alternatively, preceding high-level CMV DNAemia may lead to CMV overcoming the blood–retina barrier, entering the eye, and subsequently reactivating locally even after clearance from the blood. Further, CMV may persist in the eye for a substantial period, given that it is an immune-privileged site, before reactivating. Immunosenescence may also contribute to the late occurrence of CMVR post-SOT and explain why CMVR cases had a significantly increased median age compared with controls. Additionally, intraocular inflammation due to immune recovery uveitis can cause vision loss that is typically seen in people living with HIV (PLWH) receiving highly active antiretroviral therapy with subclinical or active CMVR and occurs due to immune reconstitution [16]. Similar inflammatory responses have also been reported in SOTr and may contribute to complications in this population [18].

CMVR has mostly been described and studied in PLWH thus far, but is becoming more prevalent in other immunocompromised individuals, including SOTr [4, 16]. Differences between HIV and non-HIV CMVR include higher male predominance and younger age, among others [14]. Comparatively, SOTr at increased risk for CMVR have been reported to be older, but no sex predominance has been identified. A retrospective cohort study by Su et al assessing clinical characteristics of CMVR in HIV versus non-HIV, showed that patients without HIV had higher mortality and recurrence rates of CMVR [19]. Although these factors were not evaluated in the present study, future and larger studies should prioritize assessing risk factors for recurrent CMVR in the SOT population.

Our study has several limitations, including the small sample size and its retrospective design. First, CMVR is a very rare diagnosis, and we were able to identify only 16 SOTr with CMVR within a province of nearly 6 million and 6000 SOTr followed during the study period. Although we identified potential CMVR cases using a database of positive CMV ocular fluid PCR results and by performing a data search of our electronic medical record system in the 2 transplant centers within our province, it is still possible we missed cases. Second, our study was not powered to assess all possible variables in multivariable analysis models. Third, given the retrospective nature of this study, we could not confirm the accuracy of the clinical diagnosis and relied on chart documentation, and for some variables, missingness could not be overcome. Fourth, although we assessed any CMV DNAemia at any time after transplant as a binary variable, and also evaluated viral load at the time of CMVR diagnosis for the cases, our analysis did not capture the full spectrum of CMV viral kinetics. As such, our ability to determine how CMV DNAemia influences long-term CMVR risk is limited. Important kinetic parameters, such as timing of onset, duration of DNAemia, and peak viral load, may influence CMVR risk. Therefore, the impact of CMV DNAemia kinetics on CMVR risk should be explored in future studies. Last, although a few of the immunosuppressant regimens used for either induction or maintenance immunosuppression were found to be used in cases at a significantly higher rate compared with controls, we were not confident that these results suggest these to be risk factors for developing CMVR post-SOT due to the small numbers.

Larger multicenter studies are needed to further study the significance of these clinical risk factors and explore other factors apart from DM. However, this is the first time a case-control study has been described to analyze the risk factors for developing CMVR post-SOT, contributing significantly to the limited existing knowledge on CMVR in SOT.

## CONCLUSION

CMVR is a rare and late complication post-SOT. DM was found to be independently associated with increased odds of developing CMVR post-SOT. Although increased age, CMV DNAemia, history of chronic heart disease, and significant lymphopenia may also be potential risk factors for CMVR, larger multicenter studies are needed to further study the significance of these clinical risk factors.
